# CO2 laser colposcopic guided surgery for the see and treat management of VHSIL: a preliminary experience

**DOI:** 10.1007/s10103-021-03413-y

**Published:** 2021-09-28

**Authors:** C. Bianchi, N. Auzzi, I. Turrini, A. De Magnis, M. G. Fallani, M. Fambrini, A. Pieralli

**Affiliations:** 1 Department of Biomedical, Experimental and Clinical Sciences, Division of Obstetrics and Gynecology, University of Florence, Largo Brambilla 3, 50134 Florence, Italy; 2 Screening Unit, Institute for Cancer Research, Prevention and Clinical Network, Florence, Italy; 3 Division of Obstetrics and Gynecology, Santo Stefano Hospital, Prato, Italy; 4 Florence, Italy

**Keywords:** Vulvar high-grade squamous intraepithelial lesion (VHSIL), CO2 laser, See and treat, HPV

## Abstract

The purpose of this study is to evaluate the efficiency of CO2 laser colposcopic guided surgery performed in an outpatient see and treat setting in the management of VHSIL. Women with a suspected diagnosis of VHSIL and no vulvoscopic suspicion of vSCC were enrolled. An electronic register of CO2 laser treatment was created where description of performing parameters (excision or ablation) was specified and personal history was recorded. Statistical analysis was performed by Fisher’s exact test. Relative risks (RR) of risk factor were calculated and expressed in odds. From September 2014 to September 2018, we enrolled a total of 63 patients who underwent CO2 laser procedure and had a minimum follow-up time of 2 years at Careggi University Hospital in Florence. Forty-eight (76.2%) patients underwent laser excision and 15 (23.8%) patients underwent ablative treatment without histological results. Undertreatment was performed in 3 cases (6.3%) with definitive histology of vSCC. Therapeutical appropriateness of CO2 laser excision was reached in 85.4% of the cases (41/48). No volunteer loss to follow-up was registered; thus, fidelity to treatment was assess at 100%. Recurrence rate within 2 years attested in 8/60 followed patients (13.3%). No personal factor was found to influence the VHSIL course. CO2 laser excision may represent an excellent therapeutic option to VHSIL because it provides adequate oncological purpose with good cosmetic and functional results and high patients’ loyalty to treatment. An expert team could allow to undergo patients with VHSIL suspicion to unique diagnostic and therapeutic procedure with significant benefits.

## Introduction

Vulvar high-grade squamous intraepithelial lesion (VHSIL) is premalignant condition and is associated with squamous cell carcinomas of the vulva (vSCC).

The incidence of VHSIL almost doubled from 1.2/100,000 in 1992 to 2.1/100,000 women in 2005, reflecting a real increased incidence related to the growing age of the population or a better recognition and more effective treatment of these precursor lesions before the development of vSCC [1].

VHSIL usually occurs in young women, in the third to the fifth decades, with the same risk factors as cervical lesions, such as number of sexual partners, smoking, and immunosuppression [2].

HPV infection is strongly associated with VHSIL, accounting for more than 80% of them, and thus, a reduction of incidence is expected worldwide by the spread in using prophylactic vaccines [3, 4].

Although spontaneous regression has been reported, VHSIL should be considered a premalignant condition; thus, treatment is considered mandatory in order to prevent vSCC since there are no screening strategies for the prevention of vulvar cancer [5].

The management of VHSIL is still not established with no clear consensus regarding the best treatment modality.

Vulvar intraepithelial neoplasia (VIN) treatment often involves multiple sites that after therapy could be afflicted by undesirable alteration of anatomy, emotional distress, and sexual problems.

Alternative treatment modalities for VHISL are classical surgical excision up to simple vulvectomy, conservative physical treatments, no invasive medical therapies as local imiquimod or cidofovir, and photodynamics [6].

Classical surgical excision conducted to 0.5–1 cm of free-disease margin in width and up to 5 mm in depth permits to obtain a specimen for histological analysis and is considered oncologically safe since the reduction of the risk of skipping vSCC whose rate is calculated to be 4–12% of the cases [7]. Furthermore, wide local excision, partial vulvectomy, and simple vulvectomy are at lower risks of early relapse in comparison to all other alternative treatments.

However, classical surgery is biased by high rates of complications such as intra- and postoperative bleeding, wound infections, scarring, psychosexual morbidity, and high operative costs for hospitalization and anaesthesia requirement.

Literature on diverse medications proposed to avoid or limit surgery in VHSIL patients lacks of evidence because of the small sample size, the difference in inclusion criteria, or the limited follow-up [6].

Cidofovir is an apoptosis enhancer of HPV-infected cells, but its use is complicated by high rates of ulceration at the site of application with unclear success in terms of cure rate which stands from 40 to 70% of treated patients [8]. Similarly, imiquimod demonstrated response rates ranging from 26 to 100% [9, 10].

Alternative, still conservative, treatment for VHSIL is photodynamic therapy which unfortunately showed numerous limits such as high rate of treatment failure, immuno-suppressive effects, poor tolerability with the need of spinal or general anaesthesia to perform it, and consequent increase of direct and non-direct costs [11].

Since litterature describes 26–30% of VHSIL relapsing within 2 years from treatment independently from the therapeutical modality [12], authors wanted to evaluate the efficiency of CO2 laser colposcopic guided surgery performed in an outpatient see and treat setting in the management of women affected by VHSIL.

## Material and method

A prospective descriptive analysis of the population referred to University Teaching Hospital of Careggi (AOUC) in Florence from September 2014 to September 2018 with a suspected diagnosis of VHSIL was conducted.

VHSIL suspicion at vulvoscopic check at Vulvar Clinic of AOUC, no vulvoscopic suspicion of vSCC at any site. At enrollment all patients were asked to read and subscribe an informed consent to CO2 laser surgical with a double diagnostic and potentially therapeutic aim.

Only patients who underwent at least one CO2 laser procedure at Colposcopic Laser Surgery Unit of AOUC and had a minimum follow-up time of 2 years entirely conducted at Vulvar Clinic of AOUC build up the case series.

Diagnosis of differentiated VIN (dVIN) at the histological sampling or vSCC was considered an exclusion criterium and referred to standard therapeutical treatment.

Data were prospectively collected in electronic medical records and retrospectively analyzed.

Every patient enrolled in the study should have recorded complete vulvoscopic evaluation with a suspected diagnosis of VHSIL at enrollement and every 6 months in the follow-up after treatment; an electronic register of CO2 laser treatment was created where description of performing parameters (excision or vaporization) was specified and personal history including personal risk factors for VHSIL such as age; smoking habit; HCV positivity; HIV positivity; number of areas with synchronous VHSIL (1, 2, more than 2); and synchronous or metachronous cervical or vaginal HPV linked pathology was recorded.

Pregnant women and those with any performance status or previous treatments were enrolled in the study since they were eligible to laser treatment.

No site or number of lesions was considered restrictive criteria to CO2 laser vulvar surgery.

Every surgical procedure was performed by CO2 laser instrument equipped with an electronic scan system (High scan surgical) and a colposcopic guided micromanipulator producing a microspot (Smart Xide2, HiScan Surgical Scanner and Easyspot Micromanipulator DEKA M.E.L.A. S.R.L., Calenzano, Firenze, Italy) ranging from 1 mm (excisional) to 12 mm (ablative); power was settled at 12 W used in a pulsing mode.

Ablative CO2 laser procedures were administered to patients affected by with a primary lesion localized in challenging anatomical sites such as clitoris or urethra or with a number of primary lesion > 2.

Treatments were conducted up to 4 mm in depth so to respect oncological purposes.

Every CO2 laser procedure was performed in an oupatient setting, under local anaesthesia; no stitches were applied after the treatment explaining to patients that the wound healing process would require from 1 to 3 weeks.

Interobserver variability was reduced by enrolling cases diagnosed by only a single expert vulvoscopist and treated by a single laser surgeon composing the vulvar diagnostic-therapeutical team.

Follow-up was every 6 months during the first 2 years after laser treatment and then annualy.

The authors wanted to asses two principles: therapeutic reliability analyzed by relapsing rate and progression rate and patients’ loyalty to treatment by the adherence rate and duration of follow-up.

Fisher’s exact test was applied to analyze numerical variables considering a statistically significative *p* < 0.05.

Relative risks (RR) of single risk factor exposure of the population were calculated and expressed in odds.

## Results

Sixty-three patients with VHSIL suspicion at vulvoscopic examination were prospectively enrolled to CO2 laser surgical procedure at Colposcopic Laser Surgery Unit with double diagnostic and therapeutic purpose.

The mean age was 52.93 years (range 20–83). The other demographic characteristics of patients are summarized in Table 1.

**Table 1:** Patients’ demographic characteristics

Demographic characteristics (63 patients)		
Mean age	52.93	20–82
Smoking	35	55%
HIV	5	8%
HPV comorbidity (vaginal or cervical)	29	46%

The mean follow-up time was 47.78 months (range 24–72).

**Fig. 1:**
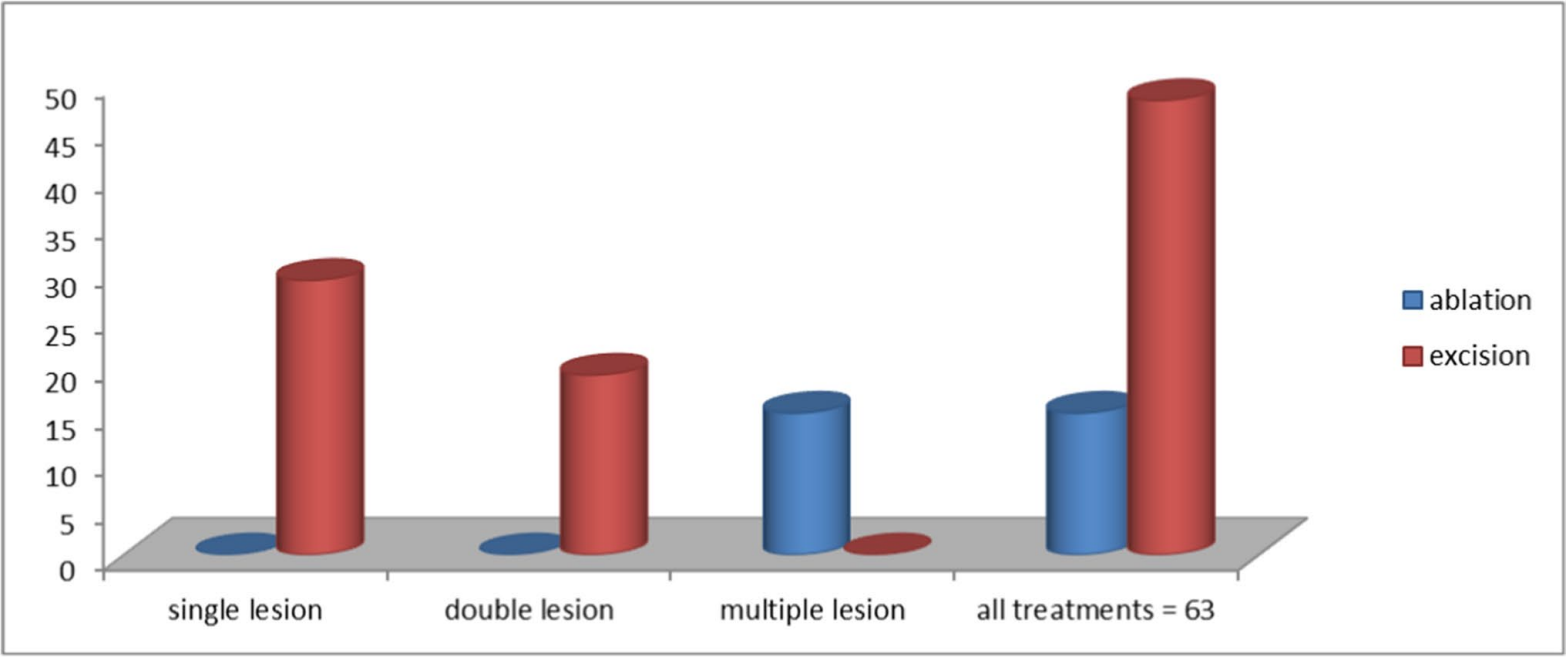
CO2 laser treatments

Forty-six percent (29/63) of patients with suspected VHSIL had associated cervical/vaginal intraepithelial neoplasia and 54% (34/63) had a multifocal lesion. Among patients with multiple site suspected VHSIL, 55.8% (19/34) had a double site and were treated by laser excision confirming VHSIL at histology analysis while 44.2% (15/34) had multiple site and underwent ablative treatment with no histological confirmation of the lesion (Fig. 1).

In total, 48/63 women (76.2%) underwent laser excision. Their mean age was 54.74 years (range 20–82), while 15/63 (23.8%) patients who underwent ablative procedures had a mean age of 49.5 years (range 23–83).

**Fig. 2:**
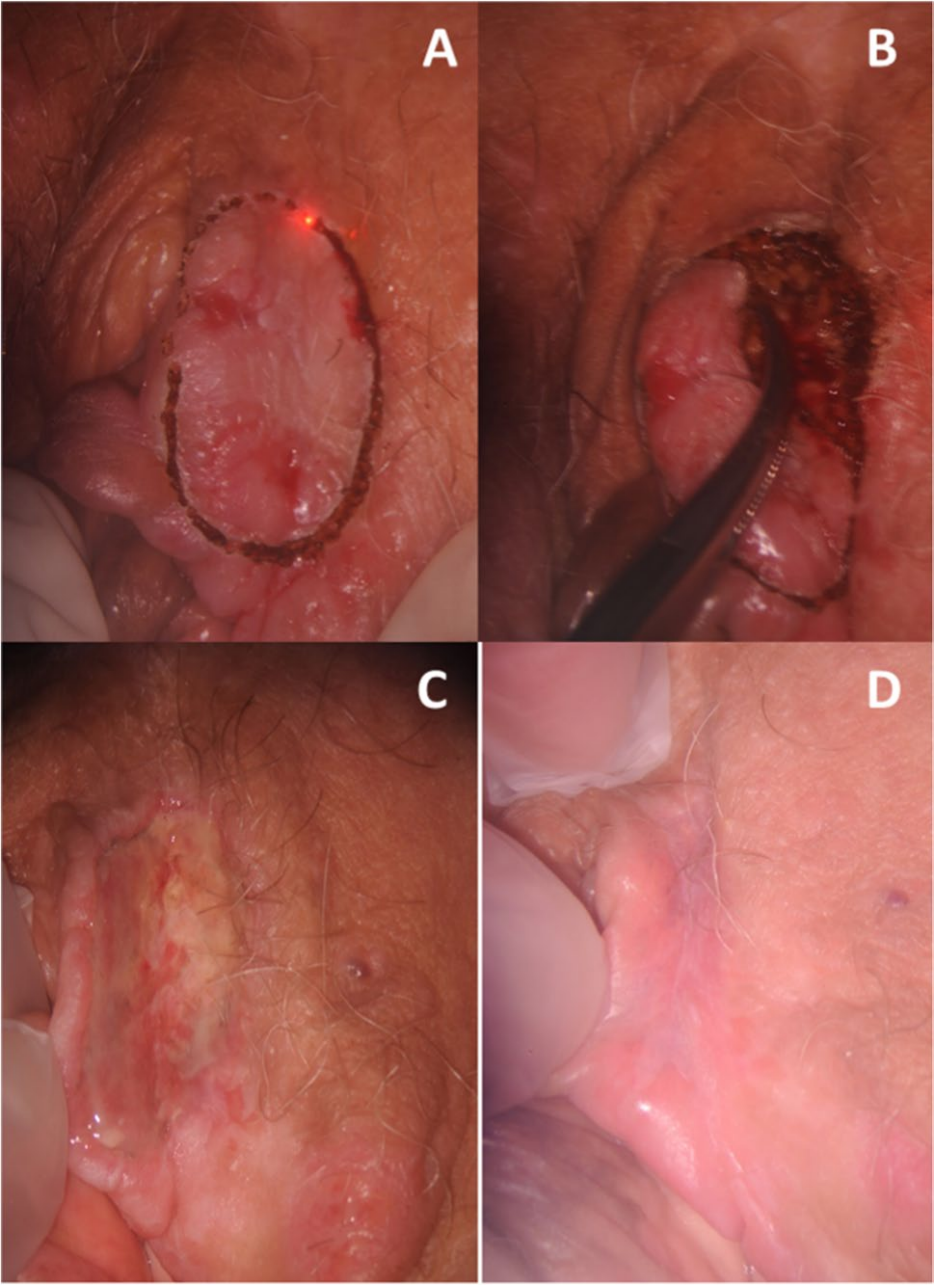
Colposcopic details of VHSIL during CO2 laser excision (A, B); follow-up visit 5 days (C) and 20 days (D) after the laser excision

No intraoperative or postoperative complications were recorded such as major bleeding, wound infection, or scarring; restitutio ad integrum was early in all patients with a mean time of 28 days (18–42 range) (Fig. 2).

No patient required hospitalization during procedure or afterward; procedures were performed totally in outpatient setting. The patients were discharged 1 h after the end of the treatment with the request to clean and moisten the wound 2 times daily with anesthetic lubrificant pomade for 1 week.

Therapeutical appropriateness of CO2 laser excision was reached in 85.4% of the cases (41/48) since definitive histology confirmed initial suspected diagnosis of VHSIL with a 100% of positive margins at the specimens; undertreatment was performed in 3 patients (6.3%) who were finally diagnosed with vSCC and 4 cases (8.3%) of definitive low grade/negative results attested overtreatment.

No volunteer loss to follow-up was registered; thus, fidelity to treatment was assessed at 100%.

In the group of excision procedures, 42.2% of women relapsed (19/45): 63.2% (12/19) were diagnosed after 2 years of primary excision, 36.8% (7/19) recurred within 2 years. All relapsing lesions underwent a second CO2 laser excisional treatment with a final histological diagnosis of VHSIL in 18/19 cases while 1 relapse progressed to vSCC.

In the group of ablation, the relapses were 4/15 (26.6%): 75% (3/4) were diagnosed after 2 years of primary treatment. All of four relapsed as single site disease and underwent CO2 laser excision. Histology confirmed suspected VHSIL in 1/4 (25%) while the other three were VLSIL (75%) (Table 2).

**Table 2:** Relapses in our series

Relapse	Ablation	Excision	*p* < 0.05 (Fisher’s test)
Total	4/15 (26.6%)	19/45 (42.2%)	n.s
Within 2 yrs	1/15 (6.6%)	7/45 (15.5%)	n.s

Total recurrence rate of VHSIL or VLSIL was 36.7% (22/60) while 1.7% of followed patients (1/60) progressed to cancer. Recurrence rate within 2 years attested in 8/60 followed patients (13.3%).

**Fig. 3:**
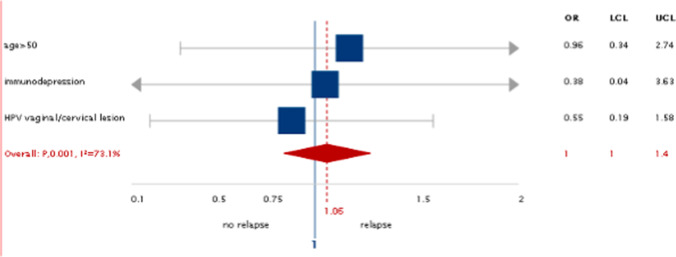
Risk factors for VHSIL relapse: no risk factor is statistically significant

No personal factor was found to influence as protective either risky the VHSIL course in the present case series (Fig. 3).

## Discussion

The aim of the treatment is to prevent development of vSCC while preserving normal vulvar anatomy and function since an increased incidence of this disease among young women and current impossibility of definitive healing.

Up to now, CO2 laser treatment was included in the group of physical conservative therapies together with argon destruction or ultrasonography tissue ablation (CUSA) reflecting limits such as oncological unsafety and high relapsing rate [13–16].

Similar to previous studies [13, 14, 17], we noted that CO2 laser surgery may represent an effective therapeutic option because it provides adequate oncological management with excellent cosmetic and functional results.

Colposcopic magnification allowed maintenance of a uniform depth throughout the sample during the excision and the ablation. The result of combining colposcopic guidance and the use of a micromanipulator to direct the beam allowed to controlled dissection of the lesions with laser excision/vaporization calibrated to a depth of 4 mm. The depth of 4 mm was able to remove entirely the epithelial layer and papillary dermis, thus respecting oncological purposes. Laser treatment can also be adjusted to size of the lesion to be treated, avoiding to treat areas of healthy tissue and thus obtaining excellent cosmetic and functional results respecting of anatomy. The anatomical integrity was also obtained thanks to the pulsing mode that respects relaxing time of the tissues and therefore reduces to non-thermal damage allowing a complete histopathological analysis and avoiding scars.

This was the explanation of why in our series although authors had 100% positive margins at the histological sample they gain a lower recurrence rate within 2 years (13.3%) than that demonstrated in the literature (30%) [18].

The authors analyzed if the lower recurrence rate within 2 years was due to a non-random selection of the sample for the main demographic and clinical risk factors for recurrence. No factors were found to be associated with development of recurrence and progression in VHSIL in the present case series. This showed that the prevalence of these risk factors in our sample was comparable to that of the general population affected by VHSIL.

To date, the goals for treatment of VHSIL should be to completely destroy the lesion, improve symptoms, exclude invasion, preserve normal vulvar anatomy and function, and avoid recurrences [19]. Excisional treatment is the preferred method because it permits histologic evaluation and detection of possible occult early invasion. Blade excision has the advantage of excluding invasion histologically, but the psychosexual morbidity, particularly in younger women, is relevant. Other authors already described the carbon dioxide laser local excision as an alternative option to preserve anatomy, but it was biased by a supposed lack in the assessment of occult invasion. The authors demonstrated that CO2 laser colposcopic guided excision is a good instrument to diagnose vSCC as first diagnosis (6.3% undertreatment rate) and in relapsing sites (1.7% of followed patients progressed to cancer). Furthermore, in the present series, no intraoperative or postoperative complications such as bleeding, wound infection, or scarring were recorded with excellent cosmetic and functional results. Instead, laser vaporization was a destructive technique and had the disadvantage of destroying the treated tissue which cannot be evaluated histologically.

Until now, any vulvar lesion found on visual inspection/vulvoscopic examination had warranted a punch biopsy to defining the therapeutic approach. The diagnosis based on punch biopsy may not be representative of the entire lesion and the risk of underdiagnosis to an occult invasive carcinoma (in our series 6.3%) [20].

This is the main reason why although laser ablation may achieve similar outcomes than laser excision; this may not be recommended due to the risk of undiagnosed cancer. Ablation can be reserved for cases of histologically proven VHSIL with multiple and widely localization.

Laser excision is required in VHSIL suspected lesions because a histological evaluation is needed to rule out possible early invasion. It is the method of choice when lesions are large because it provides sharp and clear margins eventually allowing putting stitches.

In the present study, the vulvar diagnostic-therapeutical team composed of single expert vulvoscopist and a single laser surgeon allowed to undergo patients with VHSIL suspicion to a unique diagnostic and therapeutic procedure without performing punch biopsy of every vulvar lesion; the team removed all entire lesions and obtained specimens to pathology evaluation with CO2 laser excision.

This approach called see and treat could be an innovative mean of ensuring effective, cost-efficient treatment in timely manner. This was achieved without compromising treatment success, as determined by completeness of excision of lesion. This may also represent a benefit of the reduced waiting time, with more immediate surgery preventing lesions from evolving over time.

The see and treat CO2 laser excision pathway seemed to provide a therapeutical modality that is acceptable and largely preferable by patients. In the present study, no volunteer loss to follow-up was registered indeed; thus, fidelity to treatment was assessed at 100%

Patients’ loyalty to treatment is essential because long-term post treatment follow-up is mandatory given the possibility of recurrence and the risk of progression to vulvar cancer.

Given the observed persistently increased risk of recurrence over time for these women, similar to previous studies [12, 19], the long-term surveillance is essential with a high probability of having to undergo a second treatment. The choice of a treatment that is acceptable and preferable by the patient without changing their quality of life as CO2 laser surgery is therefore necessary.

The CO2 laser procedure has no technical or clinical limitations; it can be used on every kind of patient in both ablative or excisional method.

A limitation of this surgical technique is the long learning curve that this type of surgery requires: gynecological laser surgeons must have good colposcopic skills. The laser beam is derived through a micromanipulator strictly connected to a colposcope, thus combining magnification, illumination, and precision of the colposcope with thermical characteristics of the laser.

Another important limitation of our study is the absence of a control group of patients treated with cold knife excision that is the preferred excisional procedure reported in the literature.

Recently, the ISSVD study group published a survey that showed that the literature produced to date had described VHSIL recurrence rate of up to 50% of cases with 3.8–15.1% of progression to invasive cancer whether VHSIL lesions are treated by cold knife excision, laser ablation, 5-FU, imiquimod, cidofovir, or photodynamic therapy [21].

In the same survey, it was reiterated that it was still unclear how recurrence rate differs between laser ablation and surgical excision in literature [21].

To the authors’ knowledge, the present manuscript represents the first one describing a CO2 laser excisional method on a prospective series of patient reaching comparable results to literature in term of safety and accuracy.

In conclusion, our study suggests that a vulvar diagnostic-therapeutical team can treat the lesion at the same time of diagnosis. This could have the advantage of reducing the time of expecting and the cost of hospitalization and surgery, and could permit to have a more quick diagnosis with minimum esthetic discomfort. Moreover, the authors support that CO2 laser excision may represent an excellent therapeutic option in patients with VHSIL because it provides adequate oncological purpose with good cosmetic and functional results and high patients’ loyalty to treatment.

The authors are planning to analyze aesthetical satisfaction of VHSIL patients treated by laser procedures through instruments that assess appearance-related quality of life such as multidimensional body-states relations questionnaire (MBSRQ) or EROQ0L (EQ-5D).
